# The Impact of Information Overload of E-Commerce Platform on Consumer Return Intention: Considering the Moderating Role of Perceived Environmental Effectiveness

**DOI:** 10.3390/ijerph19138060

**Published:** 2022-06-30

**Authors:** Jun Lv, Xuan Liu

**Affiliations:** Faculty of Economics and Management, East China Normal University, Shanghai 200241, China; sherryliu0730@126.com

**Keywords:** perceived information overload, online return intention, perceived environmental effectiveness, impulsive buying behavior, cognitive dissonance

## Abstract

The increasingly serious problem of consumers returning goods on e-commerce platforms has brought high costs to the Internet economy, carbon pollution to the environment, and waste of social resources. E-commerce platforms can provide useful information to assist consumers to make rational decisions, but they are often filled with useless, repetitive, and even false excessive information, which will lead to information overload and impulsive decision-making of consumers. Most of the previous literature focuses on reverse logistics, return policy, and consumer behavior tendency, etc. From the perspective of consumers’ perception of information displayed on e-commerce platforms, there are few research endeavors on the formation mechanism of perceived information overload on consumers’ return intention. Taking perceived information overload as an independent variable and consumers’ perceived environmental effectiveness as a moderation variable, this study constructs a chain mediation model that affects consumers’ online return intention. Based on the analysis of the mediating effects of impulsive buying behavior and cognitive dissonance, this study explored the moderating mechanism of consumers’ perceived environmental effectiveness on the chain mediation model. The results show that perceived information overload has a positive influence on online return intention through impulsive buying behavior, and perceived information overload has a positive influence on online return intention through cognitive dissonance. Perceived information overload also positively affects cognitive dissonance through impulsive buying behavior and thus has a significant positive chain mediating effect on consumers’ online return intention. More importantly, this research shows that consumers’ perceived environmental effectiveness can significantly moderate the chain mediation path by reducing the positive effect of the cognitive dissonance on online return intention. On this basis, this study put forward the corresponding managerial implications from the perspectives of consumers and e-commerce platforms.

## 1. Introduction

With the rapid development of Internet technology, e-commerce, and the platform economy, returning goods has gradually become a common behavior of consumers after online shopping in recent years. Returning goods has become an important part of the exchange process between enterprises and consumers, which is closely related to customer satisfaction and the maintenance of customer relationships (Ertekin, 2018) [1]. However, consumers’ returning behavior brings high costs to e-commerce platforms, extra carbon pollution to the environment, and great waste of social resources. According to *Carbon Emissions and other Environmental Impacts of Online Shopping*, a report proposed by an international environmental protection group named Greenpeace in 2017, in terms of the logistics part of return costs alone, the reverse logistics of “consumer-distribution center-warehouse” produces twice as much carbon emissions as the original transport process. According to the National Retail Federation (NRF), the amount of returned goods in the United States in 2020 alone reached $428 billion, and returned goods from online shopping more than doubled, which was the main driver of the growth of returned goods (NRF, 2021) [2]. The environmental impact of returned goods accounts for about 24% of the total environmental impact of the whole online shopping process. In contrast, the environmental impact of returned goods in physical stores only accounts for 2% of the total impact due to the low return rate (Mangiaracina et al., 2016) [3]. Bertram and Chi (2018) [4] also found that online shopping is more environmentally friendly than in-store shopping in most cases, but factors such as frequent returns and excessive packaging produce more of a carbon footprint and waste of resources. At present, most consumers are not fully aware of the environmental problems caused by returns. According to the China International Electronic Commerce Center, 65.1% of online shoppers return goods frequently; 30.6% return goods occasionally; and only 4.3% rarely return goods. In China’s “Double 11” shopping festival in 2021, the total transaction volume of e-commerce platforms was about $144.7 billion, and the total return rate was as high as 20%, among which the return rate of live streaming e-commerce was as high as 60% (Jia Li, 2021) [5], which brought extremely high economic costs to e-commerce platforms and a great waste of social resources.

Compared with the in-store shopping, the advantages of online shopping include providing attractive prices and convenient door-to-door delivery service, reducing consumers’ efforts in shopping, reducing the time spent on the journey to physical stores, and reducing the physical energy consumed by consumers in carrying products (Haridasan et al., 2018) [6]. However, unlike shopping in physical stores where products can be accessed, online shopping on e-commerce platforms often has large information asymmetry, which brings perceived purchase risks to consumers. Therefore, merchants tend to provide abundant information on platforms to improve consumers’ perception of product quality (Walsh et al., 2017) [7] so as to reduce information asymmetry on online shopping platforms. Merchants on e-commerce platforms can use Internet technology to display diversified product information in a variety of ways at a low cost. This information can be transmitted to consumers’ terminal devices through the network in the form of text, pictures, audios, videos, and live interaction. At the same time, consumers can also obtain a large amount of information related to commodities through online searches, comprehensively browse, compare, and screen the brand, and attribute and price information on commodities before making a rational purchase decision.

However, in addition to useful information that helps consumers make rational decisions, e-commerce platforms are often full of excessive useless information, including various promotional information such as “Low Price and Discount” and “Gift with Order”, as well as repeated or even false advertising information. For example, China’s “Double 11” shopping festival in 2021 had the most troublesome way to calculate discounts for shopping, with a wide variety of coupons. Different kinds of products have different discount levels, and the activities of different brands also vary greatly. Consumers often feel confused about how to take part in these activities, but buying directly can cost more than usual. If you want to use coupons well, you need to sort the goods and calculate the most cost-effective way to place an order, which wastes a lot of time and energy (Zhao Li and Yang Yinan, 2021) [8]. With the limited ability of information processing in a particular time, when consumers face a large number of brands and commodity information on e-commerce platforms, they need to search information, filter useful information, and screen out false and useless information. At this time, the amount of information is likely to exceed consumers’ information processing ability, causing them to be in a state of information overload. Repeated information or disorganized information often makes consumers miss the really useful information, or consume a lot of time in identifying the authenticity and usefulness of information, which is enough to cause them to have strong information anxiety (Hartog, 2017) [9]. At this point, consumers tend to give up rational purchasing decisions with higher costs and turn to impulsive buying behaviors (Chen, 2020) [10]. Therefore, when e-commerce platforms display excessive commodity information, it will bring information overload and high decision costs to consumers. Information overload is also likely to be the main reason for impulsive buying, cognitive dissonance, and the return of goods.

As a key issue in the field of logistics and marketing, the issue of return has been widely studied. Early research topics of the returns’ literature cover various aspects such as return policy, return logistics, inventory issues, the residual value of products, and response speed (Shen and Li, 2015; Alizadeh et al., 2020) [11,12]. With the increasing social impact of return issues, more and more e-commerce platform enterprises regard return management as an important part of corporate strategy (Hjort et al., 2013) [13], and more of the literature focuses on the behavior and intention of consumers to return goods. Lee (2014) [14] proposed that consumers’ self-cognition has a direct impact on their intention to return goods. Shang et al. (2017) [15] studied the influence of merchant policies on the formation of consumers’ return behaviors. Pei and Paswan (2018) [16] found that impulsive buying behavior leads to more returns. The research of Wachter et al. (2012) [17] found that consumers who often make impulse purchases are more likely to balance their budgets by adopting opportunistic return policies. Powers and Jack (2013) [18] discussed the possibilities of return policy leniency, switching barriers after returning goods and consumers’ opportunism tendency to influence return by affecting their cognition. Petersen and Kumar (2015) [19] explored the influence of customer satisfaction on return behavior. Seo et al. (2015) [20] found that consumers’ unplanned purchases would lead to a higher possibility of return. Lee and Yi (2017) [21] found that the policy of “gift with order” can reduce consumers’ intention to return goods by improving their perceived ownership and perceived loss of return. Pei and Paswan (2018) [16] tested the correlation between return and retail return policy as well as between return and individual moral level. Chang and Yang (2022) [22] found that both moral judgment and social consensus had a strong inhibitory effect on return behavior.

Hausman had proposed in 2000 that information overload might induce consumers to carry out irrational impulsive purchase behavior. Akram et al. (2021) [23] found that the information convenience of e-commerce platforms can improve consumers’ pragmatic purchase motivation, while insufficient information convenience can promote consumers’ hedonistic purchase motivation. Consumers’ perception of information on e-commerce platforms will influence their purchase motivation and behavior, which may affect their online return intention. However, how do consumers perceive the state of information overload on e-commerce platforms? How does perceived information overload affect consumers’ online return intention? What role does consumers’ perceived environmental effectiveness play in the influence of information overload on return intention? How can businesses design the display information of the platform so that it provides enough information for consumers to make rational decisions without causing too many returns? These problems are the main content of this paper, which are not fully explored in the current literature of return but urgently need to be solved.

Based on consumers’ perception of information, this paper constructs a chain mediation model that affects consumers’ online return intention, in which perceived information overload is the independent variable, and impulsive buying behavior and cognitive dissonance are the mediating variables. Furthermore, the moderating effect of consumers’ perceived environmental effectiveness on the chain mediation model is explored. This study collected data through questionnaires and used the empirical analysis method to do hypothesis testing. Under the support of theory and data, this paper makes an in-depth analysis of the mechanism between consumers’ perceived information overload when receiving information from e-commerce platforms and online return intention, and puts forward countermeasures and suggestions for e-commerce platforms to improve the information management and return management, reduce unnecessary waste of resources and environmental pollution, and promote sustainable development.

## 2. Conceptual Framework and Hypotheses Development

### 2.1. Mediating Effect of Impulsive Buying Behavior

Perceived information overload is a subjective description of information overload, which refers to an individual’s subjective evaluation and perception of the amount or content of information beyond processing ability.

Rook (1987) [24] defined buying impulse as a sudden strong and lasting impulse for consumers to buy something immediately. Beatty and Ferrell (1998) [25] extended this definition in their research, proposing that impulsive buying is a sudden and immediate purchase behavior. Planned buying behavior includes processes of time-consuming information search and rational decision-making (Sadikoglu, 2017) [26]. However, when making purchase decisions, the state of information overload will lead consumers to conduct complex information searches and identification, so consumers may give up rational decisions and make impulsive buying behaviors. Xiao et al. (2020) [27] also proposed in his study that impulsive buying behavior may be caused by information processing overload and decision-making difficulty. Consumers try to simplify the decision-making process of shopping by impulsive buying behavior and reduce the anxiety that may be brought by information search and processing. Therefore, perceived information overload can lead to impulsive buying behavior.

Return intention refers to consumers’ intention to return, refund, or exchange goods after purchase (Lim et al., 2016) [28]. Consumers are unlikely to take into account the consequences of impulsive buying behavior (Chen, 2020) [10]. On the one hand, when buying impulse occurs, the value of impulsive buying behavior is greater than the cost for consumers, while the cost will be greater than the benefit later, which leads to purchasing regret (Cook and Yurchisin, 2017) [29]. On the other hand, impulsive buying behavior without thinking is possible to cause consumers to overspend (Verhagen et al., 2011) [30]. When consumers calm down, regret and overspending may lead to complaints and dissatisfaction of this purchase, making it easier for consumers to have the desire to return (Lim et al., 2016) [28].

Furthermore, frequent impulse buyers are more likely to do the shopping for pleasure, which is mainly satisfied at the purchase stage (Lins et al., 2013) [31]. When the purchase is completed, consumers’ perceived value of the product decreases rapidly (Pei and Paswan, 2018) [16], and consumers are more likely to return the product.

Therefore, impulsive buying behavior will improve consumers’ online return intention. Perceived information overload may also increase online return intention by triggering impulsive buying behavior. Hence, the following hypotheses are proposed.

**Hypothesis** **1** **(H1).** *Perceived information overload positively influences impulsive buying behavior.*

**Hypothesis** **2** **(H2).** *Impulsive buying behavior positively influences online return intention.*

**Hypothesis** **3** **(H3).** *Impulsive buying behavior mediates the relationship between perceived information overload and online return intention.*

### 2.2. Mediating Effect of Cognitive Dissonance

Cognitive dissonance refers to the negative psychological state caused by inconsistent personal behavior and attitude, which was first proposed by Festinger in 1957 [32]. Product dissonance and emotion dissonance are two dimensions of cognitive dissonance (Chen et al., 2020) [10].

Wurman (1989) [33] put forward the phenomenon of “information anxiety”, indicating that individuals will be accompanied by mental and emotional fatigue in the face of massive information, and feel greater pressure, anxiety, and helplessness. Many researchers have studied the relationship between perceived information overload and the frequency of using social networks. The results prove that when social network users need to deal with a large amount of information every day, their frustration will increase, leading to user dissatisfaction and reducing user activity on social platforms (Wirth et al., 2015; Bright et al., 2015; Lee et al., 2016) [34,35,36].

Similarly, when consumers use e-commerce platforms, they are always in a state of interaction with information. When faced with excessive commodity information, consumers’ cognitive costs will increase (Jo et al., 2022) [37]. Information processing will make consumers feel anxious and frustrated, which is contrary to their original intention of shopping for pleasure. It may also reduce consumer satisfaction (Xiao et al., 2020) [27], which may lead to emotional dissonance among consumers.

At the same time, when there is too much product information, consumers will only process part of the information with limited energy, which may lead to consumers’ misjudgment of the product value (Afzal et al., 2009) [38]. After receiving the actual product, they may find that its quality is lower than expected, resulting in product dissonance. Therefore, this paper proposes that when consumers are faced with high perceived information overload, the possibility of emotional dissonance and product dissonance will be increased, and the level of cognitive dissonance will also be increased.

When consumers shop on e-commerce platforms, they may be more prone to feelings of dissonance because they are unable to touch the goods physically before purchase (Powers and Jack, 2013) [18]. After cognitive dissonance occurs, consumers usually change their cognition or behavior to relieve the inner discomfort caused by cognitive dissonance. For example, they can reduce their regret by returning goods (Zhang, 2018) [39] or withdrawing the purchase before receiving the goods (Salzberger and Koller, 2010) [40]. However, in the context of e-commerce, with more and more generous return policies, consumers begin to rely more on return behaviors to keep their psychological balance (Lee, 2015) [14].

When consumers are not satisfied with the product after purchase, they will have cognitive dissonance. Both product and emotion dissonance will promote consumers’ return intention (Chen et al., 2020) [10]. Wilkins et al. (2016) [41] and Robertson et al. (2020) [42] also found that consumers may adjust their previous decisions by returning products when they feel angry after a purchase. Hence, when consumers have cognitive dissonance, they will have a higher willingness to return goods, hoping to adjust their inner sense of imbalance by this behavior. Perceived information overload may also have an indirect effect on improving online return intention through consumers’ cognitive dissonance. Therefore, the fourth, fifth, and sixth hypotheses are proposed as follows:

**Hypothesis** **4** **(H4).** *Perceived information overload positively influences cognitive dissonance.*

**Hypothesis** **5** **(H5).** *Cognitive dissonance positively influences online return intention.*

**Hypothesis** **6** **(H6).** *Cognitive dissonance mediates the relationship between perceived information overload and online return intention.*

### 2.3. Chain Mediating Effect of Impulsive Buying Behavior and Cognitive Dissonance

Impulsive buying behavior is not planned, which may be reckless and sudden (Leong et al., 2018) [43]. Therefore, after impulsive buying, consumers may have negative emotions, thus forming a kind of post-purchase cognitive dissonance. Impulsive buying behavior can lead to product dissonance (Chen et al., 2020) [10]; for example, consumers find that they do not need the product and do not make appropriate choices before buying it (Powers and Jack, 2015) [44]. Product dissonance will also lead to emotional dissonance through inconsistent product cognition, such as consumers’ regret and disappointment for their impulsive buying behavior (Jo et al., 2022) [37]. Lim et al. (2016) [28] conducted a survey on consumers, and most respondents reported that they would feel regret and anger after impulsive buying. Lin et al. (2018) [45] concluded that consumers with impulsive buying characteristics are prone to cognitive dissonance after online shopping.

In conclusion, perceived information overload will have an impact on consumers’ impulsive buying behavior when shopping online. As impulsive buying behavior is an unplanned and reckless behavior, and as consumers are unable to contact goods before purchase when shopping online, consumers who do impulsive buying are more likely to have cognitive dissonance compared with rational buyers after receiving goods. In order to adjust this psychological imbalance, consumers may choose to return goods to relieve the inner discomfort caused by cognitive dissonance. Therefore, it is considered that:

**Hypothesis** **7** **(H7).** *Impulsive buying behavior positively influences cognitive dissonance.*

**Hypothesis** **8** **(H8).** *Impulsive buying behavior and cognitive dissonance have a chain mediating effect on the relationship between perceived information overload and online return intention.*

### 2.4. Moderating Effect of Consumers’ Perceived Environmental Effectiveness

Consumers’ perceived environmental effectiveness is an indicator to measure people’s awareness of choosing to solve environmental problems, and it reflects consumers’ judgment of their impact on the environment (Antil, 1984) [46]. Consumers with high perceived environmental effectiveness can be called green consumers, who always pay attention to the impact of their consumption on the environment (Nguyen et al., 2020) [47]. When consumers realize that consumption activities will bring adverse impacts to the environment, they will consciously make green consumption decisions (Choi and Johnson, 2019) [48]. At the same time, perceived environmental effectiveness can affect consumers’ altruism. Consumers with higher perceived environmental effectiveness can realize that the harmful impact of individual behavior on the environment is not good for others, so consumers will protect the environment through green consumption behavior (Panda et al., 2020) [49].

Return behavior also belongs to one kind of consumer behavior. More than a means to maintain customer relationships or boost profitability, return behaviors are increasingly seen as a part of corporate social responsibility as they are closely associated with environmental costs and waste of resources. For instance, environmental costs are taken into account in research related to return logistics (Kramer et al., 2015) [50]. Compared to the purchase transportation process, the carbon emissions of reverse logistics for returns double (Greenpeace, 2017). Optoro, a technology company to make retail more sustainable by eliminating all waste from returns, reported in *2021 Impact Report: Driving the Circular Revolution* that more than 27 million tons of carbon dioxide were emitted in the United States in 2021 due to returns, which is equivalent to the annual emissions of 5.9 million cars.

After the cognitive dissonance occurs, consumers usually change their cognition or behavior to relieve the feeling of discomfort (Zhang, 2018) [39]. Returning goods is a reflection of changing their behavior by canceling the decision, so as to reduce the sense of dissonance. Consumers with higher perceived environmental effectiveness are more likely to believe that their behavior will bring green benefits and are more motivated to implement green consumption behaviors (De Silva et al., 2021) [51]. Therefore, when faced with the return behavior that has environmental benefits, consumers with higher perceived environmental effectiveness will be actively aware of the negative environmental impact of implementing this behavior. In addition, they will take a positive view of their own contribution to solving this problem (Lee et al., 2014) [52], believe that their own actions can reduce the environmental problems caused by returns, and try to adjust their dissonance in other ways besides returning products (Stojanova et al., 2021) [53].

Meanwhile, consumers’ perceived environmental effectiveness moderates the chain mediation between perceived information overload and online return intention by weakening the positive effect of the cognitive dissonance on online return intention. Consumers engage in impulsive buying behavior due to information overload, and thus the individual cognitive dissonance occurs. At this point, consumers with high perceived environmental effectiveness can realize that returning goods is not environmentally friendly and believe that personal behavior can effectively contribute to the environment. Therefore, they tend to solve cognitive dissonance by adjusting their attitude toward products, rather than returning products which is not environmentally friendly to their knowledge. In consequence, the online return intention of consumers with high perceived environmental effectiveness caused by cognitive dissonance is lower than that of consumers with low perceived environmental effectiveness. In a word, the stronger the perceived environmental effectiveness of consumers, the weaker the chain mediating effect of impulsive buying behavior and cognitive dissonance. Hence, the following hypotheses are proposed:

**Hypothesis** **9** **(H9).** *Perceived environmental effectiveness negatively moderates the relationship between cognitive dissonance and online return intention. The stronger consumers’ perceived environmental effectiveness is, the weaker the impact of the cognitive dissonance on online return intention is.*

**Hypothesis** **10** **(H10).** *Through weakening the positive effect of the cognitive dissonance on online return intention, perceived environmental effectiveness moderates the chain mediating effect of impulsive buying behavior and cognitive dissonance on the relationship between perceived information overload and online return intention.*

**Figure 1:**
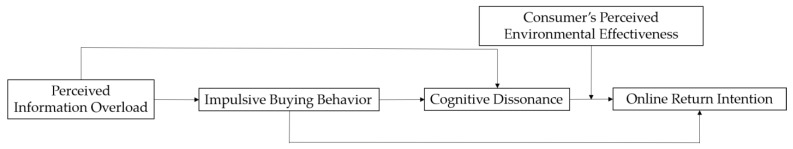
Theoretical research model.

In conclusion, this study builds a chain mediation model, which takes perceived information overload as the independent variable, online return intention as a dependent variable, and impulsive buying behavior and cognitive dissonance as mediating variables. The chain mediation path is shown as “Perceived information overload—Impulsive buying behavior—Cognitive dissonance—Online return intention” in which perceived environmental effectiveness’ moderating effect in this chain mediation is discussed. The conceptual model of this study is shown in Figure 1.

## 3. Methodology

This study adopted the method of questionnaire surveys to measure the key variables and test the model hypothesis of the obtained data. To ensure the reliability and validity of the research, all variable scales were well-established scales based on literature research and which were published in core journals and cited many times. A small-scale pre-test was conducted to test the reliability and validity of the questionnaire, and then large-scale formal questionnaires were distributed to collect research data. SPSS 23.00 (IBM, New York, NY, USA) and Mplus 8.0 (Linda Muthén & Bengt Muthén, Los Angeles, CA, USA) were used for statistical analysis of research data, and the research hypotheses and relationships between variables were tested and verified.

### 3.1. Questionnaire Design

All variables involved in this paper were measured by a Likert 7-level scale, where 1 represents “Strongly disagree”, and 7 represents “Strongly agree”.

The scale of perceived information overload was designed by referring to the research by Karr-Wisniewski and Lu (2010) [54], Zhang et al. (2016) [55], which was used to measure consumers’ perception of commodity information acquisition and processing during online shopping. This scale includes 4 items, for example, “I think a large amount of information will distract my attention”, “I often feel that the information on online shopping platforms is too much and overwhelming”, etc.

The measurement of impulsive buying behavior was designed by referring to the scale by Pei and Paswan (2018) [16], Chen et al. (2020) [10], and Özyörük (2021) [56], which measured the selective tendency of consumers in the decision-making process of compliance purchase without involving the influence of consumers’ subjective moral factors. There are 4 items in the scale, for example, “I sometimes spend more than my budget on online shopping”, “I sometimes buy things on impulse”, etc.

Cognitive dissonance was measured by referring to the research by Powers and Jack (2013) [18], Zhang (2018) [39] and Li and Choudhury (2020) [57], which involved product dissonance related to the purchased product, and emotion dissonance related to the post-purchase psychology (Zeelenberg and Pieters, 2004) [58]. The scale includes 4 items in total, for example, “After receiving the product, I sometimes find that there is a gap between it and my expectation”, “After receiving the product, I sometimes doubt whether I made a right purchase decision”, etc.

The scale of online return intention was measured by referring to the research by Chen et al. (2020) [10], which was designed from the perspectives of consumers’ attitudes, subjective norms, and perception control. There are 5 items in the scale that measured the tendency of consumers to return goods after they purchased them (Lee, 2015) [14], for instance, “Even if the return process is troublesome, I will choose to return”, “I only use the return to deal with inappropriate products purchased online”, etc.

The measurement of consumers’ perceived environmental effectiveness was measured by referring to the scale developed by Leary et al. (2014) [59], Liang et al. (2020) [60], and Panda et al. (2020) [49], which usually included consumers’ cognition of the relationship between personal behavior and environmental problems, as well as the cognition of the environmental impact of consumer behavior in a specific context. There are 4 items in total, for example, “I realize that consumer behavior affects society and the environment”, “I realize that reducing returns is environmentally friendly”, etc. All the scales and items are shown in Table A1 of Appendix A.

The original questionnaire in this paper consists of two parts. The first part is the scales of 5 core variables, including perceived information overload, impulsive buying behavior, cognitive dissonance, online return intention, and consumers’ perceived environmental effectiveness, with a total of 21 items. The second part is demographic information, including gender, age, education, and monthly income.

After the initial design and improvement of the scale, 197 valid questionnaires were collected for pre-test in this study to analyze the reliability and validity of the original questionnaire. The results showed that the internal consistency coefficient of the total scale was 0.856, and the internal consistency coefficient of each subscale was 0.924, 0.870, 0.913, 0.879, 0.856, respectively, which were all greater than 0.7 and represented good reliability. The KMO value of the total scale was 0.815; the Sig value of the Bartlett sphericity test was less than 0.001; and the total variance of factor explanation was 74.656%, which meant an acceptable result of the validity test. This study also made reasonable modifications to the questionnaire and scale items by referring to the suggestions of some subjects, to make the context easier to be understood, and finally formed the formal questionnaire.

### 3.2. Data Collection and Sample

This paper used an online platform to reach target respondents, who were mainly from mainland China, and to collect data. A total of 718 samples were finally collected during nearly one month, from 28 October 2021 to 7 December 2021. Through removing the invalid questionnaires with continuous choice of the same answer, obvious regularity of answers, too short answer time, and wrong answers to key lie test questions, 536 valid samples were obtained with a response rate of 74.65%.

In these research samples, male subjects accounted for 53.36% and female subjects accounted for 46.64%. The age of the respondents was mainly between 18 and 55 years old, accounting for 92.72%. The majority of the respondents were highly educated, among which 53.54% had a bachelor’s degree; 23.69% had a junior college degree; 7.65% had a master’s degree or above; and only 15.11% had a high school degree or below. In terms of monthly income, most of the subjects had a monthly income of 155–3116 USD, accounting for 24.07% with 155–779 USD, 47.76% with 779–1558 USD, and 14.74% with 1558–3116 USD. The specific information is shown in Table 1.

## 4. Results

### 4.1. Reliability and Validity Analysis

This research firstly tests the reliability of the scale by calculating its internal consistency coefficient, which is known as Cronbach’s Alpha. The internal consistency coefficient of perceived information overload is 0.914; the coefficient of impulsive buying behavior is 0.851; the coefficient of cognitive dissonance is 0.898; the online return intention’s coefficient is 0.882; perceived environmental effectiveness’s coefficient is 0.926; and as for the total scale, the coefficient is 0.748, with all of them are greater than the recommended value of 0.7, hence indicating the scales’ good reliability.

Secondly, this research tests the validity through exploratory factor analysis (EFA) and confirmatory factor analysis (CFA). EFA is conducted on the total scale, in which the results show that the KMO value is 0.824; bartlett’s sphericity test is significant at the 0.001 level; and the cumulative variance contribution rate is 75.456%, indicating that each variable could effectively reflect the data after factor analysis. The standardized factor loadings of both confirmatory factor analysis and exploratory factor analysis are greater than the minimum acceptable value of 0.5. None of the items need to be removed because of low factor loadings. CFA is conducted to test convergent validity and discriminant validity. The combined reliability (CR) of each variable is greater than 0.7, and the average variance extracted (AVE) is greater than 0.5, indicating that the scale had good convergent validity. The AVE of each variable was compared to the maximum share variance (MSV), which equals the square of Pearson correlations, and the AVE is found to be higher than the MSV. Therefore, all the scales have good discriminant validity. The detailed reliability and validity data are shown in Table 2.

### 4.2. Common Method Bias Tests

Since all the variables’ measurements were finished in the same questionnaire, this research tests the common method bias problems through Harman’s single factor test and CFA. Firstly, after unrotated exploratory factor analysis of all scale items, 5 factors are extracted, explaining a total variance of 75.456%. In addition, a single factor explains 16.496% of the variance, which does not exceed the maximum value of 50% and is less than half of the total variance explanation. Hence, the common method bias is considered acceptable.

In this research, CFA was conducted on key variables to test the degree of differentiation among them, and the results are shown in Table 3. According to the analysis results, the fitting indexes of the five-factor model are significantly better than other models ($\chi^{2}/df$ = 2.538, CFI = 0.968, TLI = 0.962, IFI = 0.969, RMSEA = 0.054), indicating a good discriminative validity among 5 key variables. Therefore, the common method deviation is acceptable and will not cause a serious impact on the research results.

### 4.3. Correlation Analysis

The mean value, standard deviation, and correlation coefficients of variables are shown in Table 4. The following results show that:

- Perceived information overload is positively correlated with impulsive buying behavior (r = 0.393, *p* < 0.01), cognitive dissonance (r = 0.447, *p* < 0.01), and online return intention (r = 0.167, *p* < 0.01). Meanwhile, it is negatively correlated with consumers’ perceived environmental effectiveness (r = −0.441, *p* < 0.01).
- Impulsive buying behavior is positively correlated with cognitive dissonance (r = 0.380, *p* < 0.01) and online return intention (r = 0.332, *p* < 0.01), while it is negatively correlated with consumers’ perceived environmental effectiveness (r = −0.262, *p* < 0.01).
- Cognitive dissonance is positively correlated with online return intention (r = 0.167, *p* < 0.01) and negatively correlated with consumers’ perceived environmental effectiveness (r = −0.609, *p* < 0.01).
- There is no significant correlation between online return intention and consumers’ perceived environmental effectiveness (*p* > 0.05).

The correlation analysis results above are in agreement with expectations, which preliminarily verifies the hypotheses this research proposed.

### 4.4. Mediation Model Hypothesis Test

**Figure 2:**
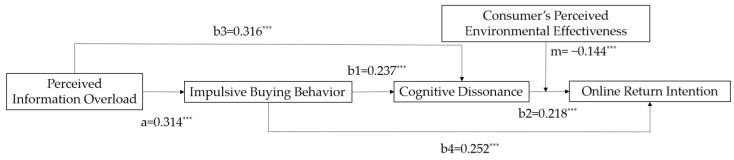
Estimation of model. Note: *** *p* < 0.01.

By using Mplus 8.0, the mediation effect of the model was tested through the Bootstrap method in this paper. The results of the theoretical model and Bootstrap test are shown in Figure 2 and Table 5.

The path value of perceived information overload on impulsive buying behavior (a) is 0.314 (*p* < 0.01), indicating a significantly positive effect from perceived information overload to impulsive buying behavior. H1 is verified. The path value between impulsive buying behavior and online return intention (b4) is 0.252 (*p* < 0.01), indicating that impulsive buying behavior has a significant positive impact on online return intention. H2 is supported. This paper also analyzed the indirect effect of perceived information overload on online return intention. The coefficient is 0.079 (*p* < 0.01), and the 95% confidence interval of Bootstrap = 5000 is (0.049, 0.119), excluding 0. Hence, H3 is verified.

The path value of perceived information overload on cognitive dissonance (b3) is 0.316 (*p* < 0.01), indicating that perceived information overload can significantly positively predict cognitive dissonance. H4 is verified. The path value between cognitive dissonance and online return intention (b2) is 0.218 (*p* < 0.01), indicating that cognitive dissonance can significantly positively impact online return intention. H5 is supported. Meanwhile, the indirect effect of perceived information overload on online return intention through cognitive dissonance is 0.069 (*p* < 0.01), and the 95% confidence interval is (0.024, 0.122), excluding 0, which means H6 is verified.

The path value between impulsive buying behavior and cognitive dissonance (b1) is 0.237 (*p* < 0.01), indicating that impulsive buying behavior has a significant positive impact on cognitive dissonance. H7 is verified. At the same time, impulsive buying behavior and cognitive dissonance play a significant chain mediating effect between perceived information overload and online return intention (β = 0.016, *p* < 0.05); the 95% confidence interval is (0.006, 0.034), excluding 0. H8 is verified.

### 4.5. Moderating Effect Hypothesis Test

Referring to the moderated chain mediation model algorithm proposed by Stride et al. (2015) [61], this paper tested the moderated effect of the model by using Mplus 8.0. The results are as follows.

**Figure 3:**
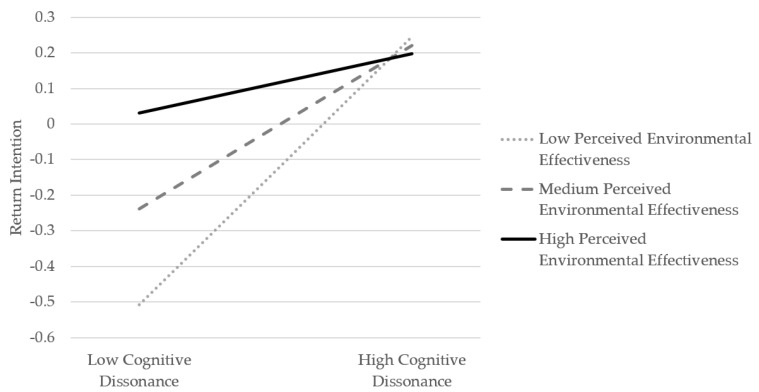
The analysis of moderating effect.

Firstly, the interaction terms of cognitive dissonance and consumers’ perceived environmental effectiveness have a significant impact on online return intention (β = −0.144, *p* < 0.01), which means that consumers’ perceived environmental effectiveness significantly moderates the relationship between cognitive dissonance and online return intention. To explain the moderator’s effect further, this research conducted a simple slope analysis by SPSS 23.0 and the output of a moderating effect diagram which is shown in Figure 3, in which the standardized mean value represents the medium level of the moderator, and the standardized mean value plus (minus) a standard deviation represents the high (low) level.

Figure 3 shows that when consumers’ perceived environmental effectiveness is at a low level, cognitive dissonance has a strong positive effect on online return intention. When consumers have a certain perceived environmental effectiveness at a medium level, the positive effect will be relatively weak. When consumers are at a strong level of perceived environmental effectiveness, the positive effect of the cognitive dissonance on online return intention will not be significant. To sum up, when consumers’ perceived environmental effectiveness becomes stronger, the positive impact of the cognitive dissonance on online return intention will gradually weaken or even become insignificant. Therefore, consumers’ perceived environmental effectiveness has a negative moderating effect between cognitive dissonance and online return intention. H9 is verified.

Then, this paper further analyzed the conditional moderated chain mediation effect. The moderated mediation effect is equal to the path values’ (a, b1) product of the interaction term (m). The test results are as follows.

In the chain mediation model proposed by this research, the product of path values and interaction terms (a × b1 × m) is −0.011 (*p* < 0.05), and the 95% confidence interval is (−0.021, −0.004), excluding 0, indicating that the chain mediation effect is moderated by consumers’ perceived environmental effectiveness.

As shown in Table 6, when consumers have a lower level of perceived environmental effectiveness, the indirect effect of perceived information overload on online return intention through impulsive buying behavior and cognitive dissonance is 0.027 (*p* < 0.01), and the 95% confidence interval is (0.011, 0.053), excluding 0, indicating that the chain mediation effect is significant. When consumers’ perceived environmental effectiveness is at the medium level, the positive indirect effect is 0.016 (*p* < 0.05), and the 95% confidence interval is (0.006,0.034), excluding 0, which means that the chain mediation effect is significant but becomes smaller. When consumers’ perceived environmental effectiveness is at a high level, the indirect effect of perceived information overload on return behavior is not significant (*p* > 0.05).

These results indicate a significantly negative moderating effect of consumers’ perceived environmental effectiveness on the chain mediation effect of impulsive buying behavior and cognitive dissonance. When consumers’ perceived environmental effectiveness is higher, the indirect effect of perceived information overload on online return intention will be significantly weakened. H10 is verified. Results of hypothesis testing are presented in Table 7.

## 5. Discussion and Implications

### 5.1. The Mediating Effect of Impulsive Buying Behavior and Cognitive Dissonance

This study found that perceived information overload positively affected online return intention through impulsive buying behavior, which verified the mediating effect of impulsive buying behavior. Firstly, perceived information overload has a significant positive impact on consumers’ impulsive buying behavior, which is verified by H1. When the high degree of perceived information overload occurs, consumers are more likely to be mad about the information on the Internet, relative to other consumers, and the information search and processing costs will be higher. Therefore, in the face of rich information on e-commerce platforms, consumers may give up time-consuming rational purchase decisions and instead make impulsive purchases. Secondly, H2 verifies that impulsive buying behavior has a significant positive impact on online return intention. Impulsive purchases stimulated by external factors can bring to consumers a temporary sense of pleasure, and the purchase decision made in an excited state may make consumers spend more than the budget, which will bring consumers regret later, leading to a decrease in the value of the shopping experience, and thus the return intention. Finally, H3 verifies the mediating effect of impulsive buying behavior between perceived information overload and online return intention; that is, perceived information overload will promote consumers to carry out impulsive buying behavior and then improve online return intention.

Perceived information overload affects online return intention through cognitive dissonance, which verifies the mediating effect of impulsive buying behavior. First, H4 verifies that perceived information overload has a significant positive impact on cognitive dissonance. The information processing capacity of consumers is limited, and a large amount of commodity information turns consumers into a state of information overload, resulting in information anxiety, user dissatisfaction, and emotional dissonance of consumers. The increase in the cost of information processing may also make consumers make errors in the process of identifying information, resulting in product dissonance. Secondly, H5 verifies that cognitive dissonance has a significant positive impact on online return intention. After cognitive dissonance occurs, consumers usually change their existing cognition or behavior to balance their inner dissonance. Online shopping usually provides consumers with low-cost or no-cost return policies, so consumers increasingly rely on a return to deal with cognitive dissonance. Finally, H6 verifies the mediating effect of cognitive dissonance between perceived information overload and online return intention; that is, perceived information overload will improve consumers’ level of cognitive dissonance, thus improving their online return intention.

H7 and H8 verify the chain mediating effect of impulsive buying behavior and cognitive dissonance. Perceived information overload has a significant positive indirect effect on consumers’ online return intention through impulsive buying behavior and cognitive dissonance. When faced with overwhelming information and numerous product choices on e-commerce platforms, consumers with high perceived information overload will feel that the information processing capacity required to make rational purchase decisions is beyond their capacity, so they will choose irrational impulsive purchase behavior to reduce the information processing cost. Under the influence of perceived information overload, the possibility of impulsive buying behavior is increased, and finally the possibility of post-purchase cognitive dissonance is increased, thus improving consumers’ online return intention.

Most of the previous studies believe that online shopping platforms have a lot of information asymmetry, which requires merchants to provide a lot of information on the platform to reduce information asymmetry. However, this study found that excessive information could not only improve information asymmetry, but also lead to more impulsive buying behaviors, higher cognitive dissonance, and more frequent returns by consumers. When consumers are faced with the volume of information on e-commerce platforms, they may feel overwhelmed and suffer from perceived information overload. Therefore, consumers tend to give up rational purchase decisions with high information processing costs and turn to impulsive buying behaviors, resulting in post-purchase cognitive dissonance and return intention. In conclusion, e-commerce platforms and merchants should pay attention to the information processing problems of consumers in online shopping and reduce the influence of perceived information overload on online return intention through impulsive buying behavior and cognitive dissonance.

First of all, consumers should be provided with sufficient, concise, clear, and comprehensive information, so as to meet their information needs directly and quickly and reduce the information asymmetry of e-commerce platforms. At the same time, it is necessary to reduce unnecessary repetitive information and eliminate all false information to reduce the cost of information screening and authenticity identification for consumers. Second, consumers face a rich variety of goods; it takes more energy to compare information and select products. E-commerce platforms and merchants can use optimized recommendation algorithms to provide consumers with accurate and personalized recommendation services, reduce the decision-making cost, support rational decisions, and further reduce the possibility of impulse buying behavior. In addition, some promotion activities such as video and livestreaming are relatively hot marketing models in the e-commerce industry, which can better show product information to consumers and understand consumers’ doubts and confusion through real-time online communication. In this way, e-commerce platforms can provide accurate information that meets consumers’ personalized needs to assist them to make rational decisions. Finally, the rules and information of promotional activities can be simplified and disclosed in time. It is also important that the specific content of rules and information should be clarified. For example, relevant terms and the specific use of the scope of discounts should be actively explained to consumers to reduce unnecessary complex calculations. All the above measures can make consumers quickly obtain clear and sufficient promotion and discount information, which not only promotes sales, but also promotes rational and calm purchasing behavior of consumers, thus reducing cognitive dissonance and return behavior after purchases.

### 5.2. The Moderating Effect of Perceived Environmental Effectiveness

This study explores the influence of different degrees of consumers’ perceived environmental effectiveness on the formation process of online return intention. H9 verifies that consumers’ perceived environmental effectiveness significantly moderates the positive impact of the cognitive dissonance on online return intention. Consumers’ perceived environmental effectiveness can negatively interact with consumers’ post-purchase cognitive dissonance and weaken the positive impact of cognitive dissonance on online return intention. When consumers’ perceived environmental effectiveness level is low, cognitive dissonance has a greater impact on online return intention, and consumers are more likely to carry out the return behavior. When the level of consumers’ perceived environmental effectiveness becomes higher, the positive impact of cognitive dissonance on the online return intention will gradually weaken, or even become insignificant, and consumers will not be inclined to use return behavior to adjust cognitive dissonance. In conclusion, the level of consumers’ perceived environmental effectiveness can influence consumers’ cognition of environmental protection of return behavior so as to change their tendency of choosing ways to adjust their inner dissonance, thus weakening the influence of post-purchase cognitive dissonance on online return intention.

H10 verifies that consumers’ perceived environmental effectiveness has a significant moderating effect on the chain mediation path of “perceived information overload—impulsive buying behavior—cognitive dissonance—online return intention”. The indirect effect of perceived information overload on online return intention through the chain mediation of impulsive buying behavior and cognitive dissonance is moderated by consumers’ perceived environmental effectiveness. When consumers’ perceived environmental effectiveness level is low, perceived information overload has a significant indirect effect on online return intention, and the chain mediating effect of impulsive buying behavior and cognitive dissonance is significant. With the increase in consumers’ perceived environmental effectiveness, this indirect effect will gradually weaken. When the perceived environmental effectiveness is increased to a certain level, the indirect effect of perceived information overload on online return intention is not significant. Even if consumers are in a state of information overload, the online return intention caused by impulsive buying behavior and cognitive dissonance may not be increased. The enhancement of consumers’ perceived environmental effectiveness significantly weakened the mediating effect of cognitive dissonance and the chain mediating effect of impulsive buying behavior and cognitive dissonance significantly and further reduced consumers’ return intention after impulsive purchases. As a result, it is essential to cultivate consumers’ perceived environmental effectiveness, improve consumers’ environmental awareness, and strengthen consumers’ understanding of the fact that the return behavior is not environmentally friendly.

Perceived environmental effectiveness education is a part of environmental education, with the characteristics of national and lifelong, which means that the government plays a vital role in it. The government can call for communities and other organizations to carry out educational activities such as environmental knowledge popularization activities to disseminate environmental knowledge about returns to the public. At the same time, the government can also enlarge the coverage of return-relevant environmental education through the publicized public welfare advertisement, thus enhancing the effect of the perceived environmental effectiveness education. For the platform merchants, merchants can insert environmental protection factors into the design of the online store page so that consumers can be subtly influenced in the process of browsing the page. Merchants can also popularize the environmental protection issues involved in forward and reverse logistics of online shopping to consumers in their daily operations. For example, combining environmental education with e-commerce live-streaming is a new way worth exploring.

Besides the promotion of government and merchants, environmental awareness education also needs the active participation and promotion of consumers. Consumers are in closer contact with each other, and in many cases, word of mouth is more effective than serious scientific courses. Community and other departments can guide consumers to exchange environmental protection knowledge to achieve effective environmental education among a small range of consumer groups and improve the popularization rate of environmental awareness education. Meanwhile, the publicity effect of key opinion leaders (KOL) and key opinion consumers (KOC) in the Internet era should be made good use of. The development of we media has given birth to a large number of KOLs and KOCs. These KOLs and KOCs have a great influence and voice among their fans compared with other consumers. These consumers can export environmental protection knowledge to their fans by making videos and publishing articles, popularizing the disadvantages of unnecessary return behavior, and realizing the education of environmental awareness among consumers.

## 6. Conclusions and Further Research

The problem of returns is becoming more and more serious, which brings high cost to platforms and great waste of social resources. In the past, most of the research on online shopping return focused on reverse logistics, return policy, and consumer behavior tendency. Different from previous studies, this paper extends the research perspective of influence factors of online return intention. From the perspective of the consumers’ information perception, this research explores the influence of perceived information overload on consumers’ online return intention through a chain mediation model, which is constructed with impulsive buying behavior and cognitive dissonance as mediating variables. The moderating effect of consumers’ perceived environmental effectiveness on the chain mediation model is also discussed.

There are still some limitations in this paper that need to be further solved in future research. The objects of this paper are consumers, but e-commerce platforms and merchants were not investigated, and the categories of goods were not distinguished during the survey process. Therefore, future research can focus on the following aspects:

Firstly, this paper adopts the method of a consumer survey to collect data during the research process, and the data collected are about consumers’ perception in the process of online shopping without carrying out research on merchants on the platform. Therefore, further research can be carried out in this aspect in the future to explore whether the different perceptions between the perspectives of merchants and consumers will have different impacts on online return intention. Additionally, further research also can consider the impacts of the demographic characteristics of the sample.

Secondly, the categories of goods purchased by the subjects were not discussed in the survey. In actual business activities, the return policies of functional products (for example, daily cosmetics) and innovative products (for example, electronic products) may differ greatly. Consumers’ information overload perception, purchase behavior, post-purchase cognition, and online return intention may also vary with the change in the product category. Therefore, further research in the future can explore how the influence mechanism of consumers’ online return intention will change in different types of commodity markets.

Thirdly, this study studies the influence mechanism of excessive returns in e-commerce platform activities from consumers’ perception of information. This influence path is realized through the chain mediating effect of impulsive buying behavior and cognitive dissonance. Future research can further explore how e-commerce platforms, merchants, and consumers play a role together and study the more comprehensive impact path of online return intention to improve the system of platform, merchants, and consumers to participate jointly in the suppression of returns and provide more theoretical support and practical inspiration for the sustainable development of the e-commerce industry.

## Figures and Tables

**Table 1:** Descriptive statistics.

Items		Frequency (Percent)
Gender	Male	286 (53.36%)
	Female	250 (46.64%)
Age	Younger than 18	3 (0.56%)
	18–25	58 (10.82%)
	26–35	96 (17.91%)
	36–45	232 (43.28%)
	46–55	111 (20.71%)
	Older than 55	36 (6.72%)
Education	High school degree or below	81 (15.11%)
	Junior college degree	127 (23.69%)
	Bachelor’s degree	287 (53.54%)
	Master’s degree or above	41 (7.65%)
Monthly Income	Less than 76 USD	12 (2.24%)
	76–155USD	31 (5.78%)
	155–779 USD	129 (24.07%)
	779–1558 USD	256 (47.76%)
	1558–3116 USD	79 (14.74%)
	More than 3116 USD	29 (5.41%)

**Table 2:** Reliability and Validity Analysis Results.

Variables	Items	EFA Loadings	CFA Loadings	Cronbach’s Alpha	KMO	CR	AVE	MSV
PIO	PIO1	0.840	0.873	0.914	0.855	0.915	0.728	0.200
	PIO2	0.828	0.830					
	PIO3	0.874	0.832					
	PIO4	0.868	0.877					
IB	IB1	0.782	0.688	0.851	0.803	0.852	0.592	0.155
	IB2	0.816	0.742					
	IB3	0.765	0.779					
	IB4	0.831	0.859					
CD	CD1	0.810	0.878	0.898	0.845	0.896	0.682	0.371
	CD2	0.784	0.792					
	CD3	0.824	0.812					
	CD4	0.781	0.821					
RI	RI1	0.852	0.831	0.882	0.870	0.886	0.609	0.110
	RI2	0.725	0.681					
	RI3	0.829	0.788					
	RI4	0.832	0.804					
	RI5	0.835	0.790					
PEE	PEE1	0.881	0.911	0.926	0.856	0.925	0.757	0.371
	PEE2	0.828	0.838					
	PEE3	0.824	0.865					
	PEE4	0.845	0.864					
**Total**				0.748	0.824			

Note: PIO, Perceived information overload; IB, Impulsive buying behavior; CD, Cognitive dissonance; RI, Online return intention; PEE, Perceived environmental effectiveness.

**Table 3:** Confirmatory factor analysis results.

Model	$\chi^{2}/df$	CFI	TLI	IFI	RMSEA
Five-Factor Model: PIO, IB, CD, RI, PEE	2.538	0.968	0.962	0.969	0.054
Four-Factor Model: PIO, IB + CD, RI, PEE	5.952	0.895	0.876	0.895	0.096
Three-Factor Model: PIO + IB + CD, RI, PEE	8.640	0.839	0.809	0.840	0.120
Two-Factor Model: PIO + IB + CD + RI, PEE	11.473	0.780	0.738	0.781	0.140
One-Factor Model: PIO + IB + CD + RI + PEE	15.499	0.703	0.637	0.704	0.165

Note: PIO, Perceived information overload; IB, Impulsive buying behavior; CD, Cognitive dissonance; RI, Online return intention; PEE, Perceived environmental effectiveness.

**Table 4:** The matrix of descriptive statistics and correlation.

	Mean	SD	1	2	3	4	5
PIO	4.889	1.553	(0.853)				
IB	5.047	1.287	0.393 **	(0.770)			
CD	4.979	1.398	0.447 **	0.380 **	(0.826)		
RI	4.883	1.315	0.167 **	0.332 **	0.167 **	(0.781)	
PEE	3.922	1.615	−0.411 **	−0.262 **	−0.609 **	−0.047	(0.870)

Note: N = 536; ** *p* < 0.05; the values in parentheses are the square roots of AVE. Below them are the Pearson correlations between the constructs’ values. PIO, Perceived information overload; IB, Impulsive buying behavior; CD, Cognitive dissonance; RI, Online return intention; PEE, Perceived environmental effectiveness.

**Table 5:** Mediating effect and 95% confidence interval estimated by Bootstrap method.

Path		Indirect Effect Estimation	*p*-Values	CI at 95% Level	
**Total indirect effect**		0.164	0.000	0.106	0.232
**Indirect effect**	PIO-IB-RI	0.079	0.000	0.049	0.119
	PIO-CD-RI	0.069	0.005	0.024	0.122
	PIO-IB-CD-RI	0.016	0.017	0.006	0.034

Note: PIO, Perceived information overload; IB, Impulsive buying behavior; CD, Cognitive dissonance; RI, Online return intention.

**Table 6:** The analysis of moderated chain mediation effect.

Conditional Moderator	Path: PIO-IB-CD-RI			
	Indirect Effect	*p*-Values	CI at 95% Level	
			Lower Limit	Upper Limit
Low PEE	0.027	0.009	0.011	0.053
Medium PEE	0.016	0.017	0.006	0.034
High PEE	0.005	0.262	−0.003	0.017

Note: PIO, Perceived information overload; IB, Impulsive buying behavior; CD, Cognitive dissonance; RI, Online return intention; PEE, Perceived environmental effectiveness.

**Table 7:** Results of hypothesis testing.

Hypotheses	Results
Hypothesis 1 (H1).	Supported
Hypothesis 2 (H2).	Supported
Hypothesis 3 (H3).	Supported
Hypothesis 4 (H4).	Supported
Hypothesis 5 (H5).	Supported
Hypothesis 6 (H6).	Supported
Hypothesis 7 (H7).	Supported
Hypothesis 8 (H8).	Supported
Hypothesis 9 (H9).	Supported
Hypothesis 10 (H10).	Supported

## Data Availability

Data are available on reasonable request.

## Appendix A

**Table A1:** Survey on Online Return Intention.

Scales	Items
Perceived Information Overload	I think a large amount of information will distract my attention. 1 (strongly disagree) to 7 (strongly agree)
	I often feel that the information on online shopping platforms is too much and over-whelming. 1 (strongly disagree) to 7 (strongly agree)
	I often feel like there’s more information on the platform than I can handle. 1 (strongly disagree) to 7 (strongly agree)
	I often find that there are so many choices that I don’t want to make the effort to compare and choose. 1 (strongly disagree) to 7 (strongly agree)
Impulsive Buying Behavior	I sometimes spend more than my budget on online shopping. 1 (strongly disagree) to 7 (strongly agree)
	I sometimes buy things on impulse. 1 (strongly disagree) to 7 (strongly agree)
	Sometimes I want to buy something when I see a picture of a product. 1 (strongly disagree) to 7 (strongly agree)
	I sometimes have a sudden urge to buy something, even if it’s not in my shopping plan. 1 (strongly disagree) to 7 (strongly agree)
Cognitive Dissonance	After receiving the product, I sometimes find that there is a gap between it and my expectation. 1 (strongly disagree) to 7 (strongly agree)
	After receiving the product, I sometimes doubt whether I made a right purchase decision. 1 (strongly disagree) to 7 (strongly agree)
	After receiving the product, I wondered if I really needed it. 1 (strongly disagree) to 7 (strongly agree)
	After receiving the product, I would wonder if the merchant was making false claims. 1 (strongly disagree) to 7 (strongly agree)
Online Return Intention	Even if the return process is troublesome, I will choose to return. 1 (strongly disagree) to 7 (strongly agree)
	I only use the return to deal with inappropriate products purchased online. 1 (strongly disagree) to 7 (strongly agree)
	As long as the product doesn’t fit, I intend to return it. 1 (strongly disagree) to 7 (strongly agree)
	Even if I buy something cheap, I will choose to return it. 1 (strongly disagree) to 7 (strongly agree)
	I think it is the right decision to return the unsuitable product. 1 (strongly disagree) to 7 (strongly agree)
Perceived environmental effectiveness	I realize that consumer behavior affects society and the environment. 1 (strongly disagree) to 7 (strongly agree)
	I realize that reducing returns is environmentally friendly. 1 (strongly disagree) to 7 (strongly agree)
	I believe I can make a contribution to the solution of environmental problems. 1 (strongly disagree) to 7 (strongly agree)
	I believe that reducing returns can have a positive impact on the environment. 1 (strongly disagree) to 7 (strongly agree)
